# Beneficial effects of dog fur as a potting soil component

**DOI:** 10.17912/micropub.biology.001775

**Published:** 2025-11-21

**Authors:** Zoe A Long, Benjamin S Ramage

**Affiliations:** 1 Biology, Randolph–Macon College, Ashland, Virginia, United States

## Abstract

Commercial potting soil mixes are typically dominated by peat moss, a nonrenewable resource. Here, we explore the potential of a novel alternative growth medium: dog fur waste collected from retail grooming salons. We evaluated three common container-grown crops (basil, lettuce, and marigold) and found that all three exhibited substantially improved growth when a standard potting mix was amended with dog fur (25% or 50% by volume). These surprising effects encompassed multiple response variables, including both vegetative and reproductive structures. Our results suggest that dog fur has potential for a wide range of applications spanning both edible and ornamental plant production.

**Figure 1. Effects of dog fur on plant growth:**
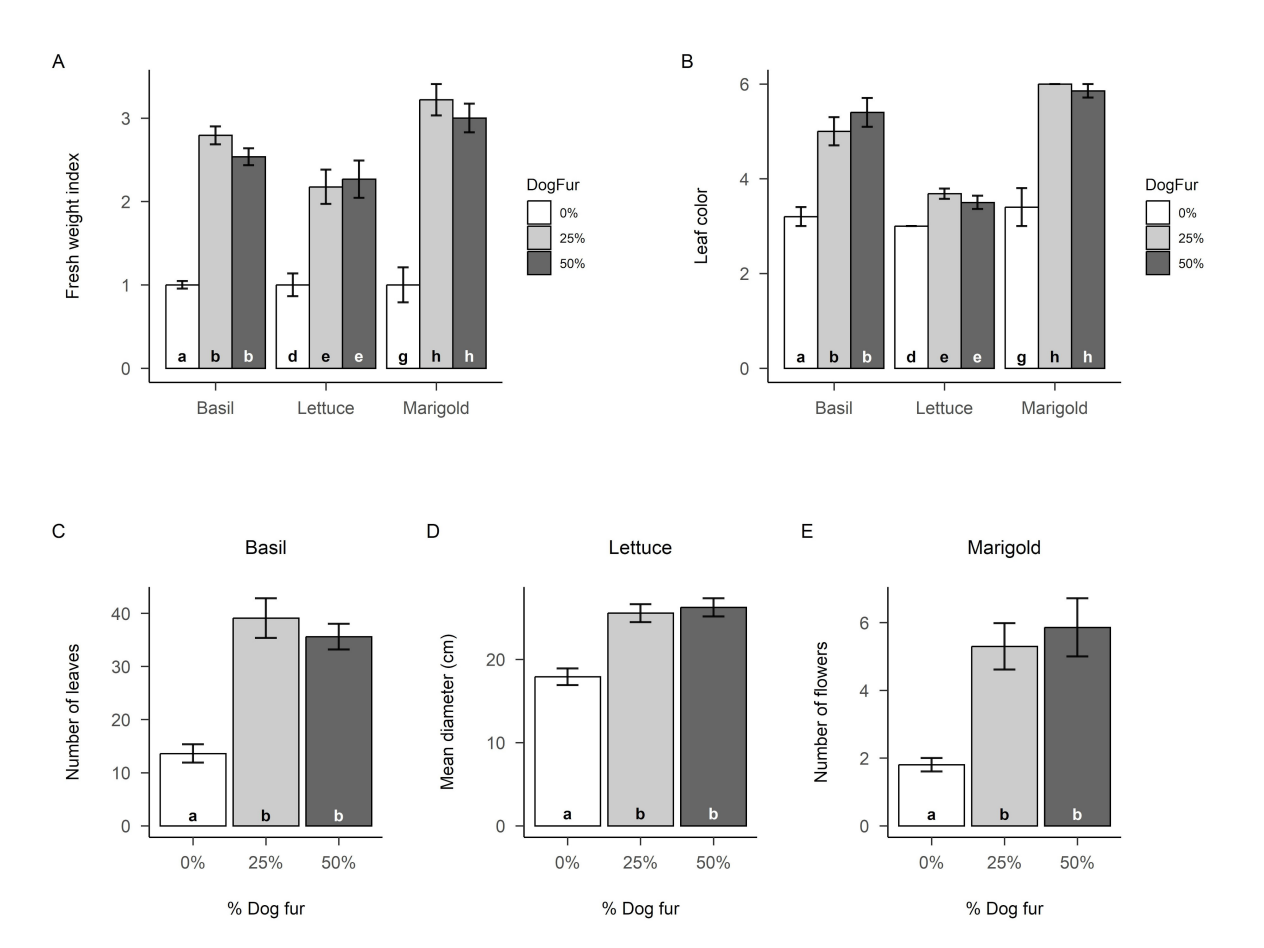
A) fresh weight, B) leaf color, C) basil leaf number, D) lettuce plant diameter, and E) marigold flower count (open flowers + flower buds). Error bars represent SE. Lower-case letters at the bottom of each column indicate results of Tukey HSD tests, which were done separately for each species. Fresh weight index = fresh weight values standardized to the mean of the control group (0% fur) for each species, to facilitate direct comparison across species.

## Description

The agricultural industry uses various animal waste products as fertilizers and soil amendments for plant growth (Alao et al. 2017; Girotto and Cossu 2017). While manure is most commonly utilized, there are other widely available waste products that have yet to be explored. For example, large quantities of fur from domestic dogs ( *Canis familiaris* ) are generated by pet grooming salons. In the United States alone, approximately 60 million households own at least one dog (Applebaum et al. 2023), and there are an estimated 8,000 pet grooming businesses, including both independent operations and large companies (American Pet Products Association 2020).

Dog fur has yet to be investigated in an agricultural context, but all hair-like structures are chemically similar (Senthilkumar et al. 2023), and both sheep wool and human hair have been studied. Wool has been shown to increase plant growth when incorporated into field soil (Zheljazkov et al. 2008) and container-based potting mixes (Górecki and Górecki 2010; Zheljazkov et al. 2009), with concurrent nutrient increases in soil and/or plant tissue (Zheljazkov et al. 2008; Zheljazkov et al. 2009). Similarly, several studies (reviewed in Gupta 2014) have found agricultural benefits of human hair, including increased growth and higher plant tissue nutrient concentrations.

In container-based production, most potting mixes are dominated by peat moss, which provides numerous advantages including near optimal bulk density and water-holding capacity (Schmilewski 2008; Stevenson 1974). However, peat extraction is environmentally unsustainable due to its slow regeneration rates, and there are numerous additional ecological concerns (Kumar 2017). Many materials have been investigated as sustainable potting soil alternatives, including sheep wool, which has a water-holding capacity similar to that of peat moss (Górecki and Górecki 2010).

The objective of this study was to investigate the potential of a novel alternative growth-medium for container-grown crops: dog fur, which may provide structural attributes comparable to peat moss while simultaneously supplying some essential nutrients. We evaluated three crops commonly grown in containers (basil, *Ocimum basilicum* ; lettuce, *Lactuca sativa* ; and French marigold, *Tagetes patula* ), growing each in three dog fur concentrations (0%, 25%, and 50% dog fur by volume, mixed into a standard commercial potting mix). In addition, to determine if surface contaminants (e.g. shampoo) influence growth, we incorporated a rinsing treatment (three sequential soakings).

Plants were allowed to grow until the largest individuals of each species began to outgrow their pots, at which point two variables (fresh weight and leaf color) were recorded for all three species, along with some species-specific variables. To facilitate comparison across crops, fresh weight values were standardized to the mean of the control group (0% fur) for each species. The resulting fresh weight index thus has a mean of 1.0 for each control, and other values can be readily interpreted in relation to the control.

Our results reveal that plants thrive when grown in potting soil that contains dog fur. With all species pooled, fresh weight was approximately 2.5 times greater in the 25% and 50% dog fur treatments than in the 0% dog fur control (25% vs 0%: p<0.001, effectCI=1.14-2.06; 50% vs 0%: p<0.001, effectCI=1.04-2.00), but there was no difference between the 25% and 50% fur treatments (p=0.877, effectCI=-0.48-0.32) (ANOVA p<0.001, F=38.96, df=2,87). Similarly, with all species pooled, leaf color was higher (darker green) for both fur treatments compared to the control (25% vs 0%: p<0.001, effectCI=0.76-2.17; 50% vs 0%: p<0.001, effectCI=0.76-2.23), but there was no difference between 25% and 50% fur (p=0.993; effectCI=-0.59-0.65) (ANOVA p<0.001, F=14.67, df=2,87). Species-specific analyses support these broad findings, with each species individually displaying the same qualitative trends (all p-values comparable), but with some interesting differences in effect size ( Fig. 1A, 1B). Dog fur (25% or 50%) yielded basil fresh weight about 2.5 times greater than the control, while approximately doubling lettuce fresh weight, and approximately tripling marigold fresh weight ( Fig. 1A ). Leaf color displayed similar patterns, with lettuce exhibiting a small but significant difference between the control and fur treatments, and both basil and marigold exhibiting noticeably darker leaves when fur was incorporated into the potting soil ( Fig. 1B ).

Species-specific response variables exhibited the same general pattern: clear benefits of dog fur, but with no difference between the 25% and 50% treatments. This trend occurred for basil height and leaf number, lettuce diameter and leaf number, and marigold flower count. The only exception was marigold plant height, which was unaffected. Commercially relevant responses to the addition of dog fur include a greater than 150% increase in the number of basil leaves ( Fig. 1C ), a nearly 50% increase in lettuce plant diameter ( Fig. 1D ), and an approximate tripling of marigold flowers ( Fig. 1E ).

Rinsing the dog fur had no effect on its potential as a potting soil component. When all three species were pooled for analysis, fresh weight did not differ between rinsed and unrinsed treatments (p=0.308, effectCI=-0.64-0.15), and both treatments yielded heavier plants than the no-fur control (rinse vs no-fur: p<0.001, effectCI=0.99-1.91; unrinsed vs no-fur; p<0.001, effectCI=1.23-2.16) (ANOVA p<0.001, F=40.79, df=2,87). Similarly, leaf color was unaffected by rinsing (p=0.858, effectCI=-0.48-0.75), and both treatments produced darker leaves than the control (rinse vs. no-fur: p<0.001, effectCI=0.83-2.23; unrinsed vs no-fur; p<0.001, effectCI=0.69-2.13) (ANOVA p<0.001, F=14.85, df=2,87). When species were analyzed individually, all response variables were similarly unaffected by rinsing (all p-values > 0.05).

Overall, our results tell a very clear story: plants love dog fur. All three species exhibited substantially higher fresh weights when fur was incorporated into the soil, and similar effects also occurred across several additional variables. In addition, dog fur consistently produced darker green leaves than the corresponding controls for each species, suggesting that the fur may have increased nitrogen availability (Fayez et al. 2022). Nitrogen is vital for many growth and development processes (Anas et al. 2020), and previous research has found that sheep wool and human hair can increase nitrogen levels in the soil as well as in plant tissue (Gupta 2014; Zheljazkov et al. 2008).

Peat moss is a non-renewable resource that is being slowly but steadily exhausted from high-latitude peat bogs (Kumar 2017; Schmilewski 2008). In addition, peat must be transported long distances and can account for a considerable proportion of production costs (Kumar 2017). Accordingly, it would have been exciting to find that a free, readily available waste product could produce outcomes that are simply comparable. This was all we reasonably hoped for initially, and thus we were astounded to find that our 25% and 50% dog fur mixtures dramatically outperformed the control (a commercial potting mix composed of approximately 50% peat moss). We also found that rinsing the fur had no detectable effect, suggesting that growers could opt to skip rinsing (avoiding the associated labor) or to include a rinsing routine (to satisfy consumers and/or to remove potential contaminants on the fur).

We do not know how our dog fur mixtures would compare when a liquid feeding (or other fertilization) regime is incorporated, as would typically be the case for commercial growers. It might be that the benefits disappear, but even if this is the case, our results demonstrate not only that peat can potentially be replaced by a free, renewable resource (fur), but also that dog fur effectively comes with its own fertilizer, reducing costs and potential environmental impacts (e.g. runoff leading to eutrophication; Withers et al. 2014).

To fully evaluate the utility of dog fur waste as a peat moss alternative, several additional issues should be explored. For instance, further experimentation will be necessary to determine chemical and microbial composition as well as water-holding capacity (moisture retention seemed roughly comparable when dog fur was incorporated, although we did not formally collect data). Analyses such as these should help to clarify exactly why plants seem to thrive in potting soil amended with dog fur, and will also be necessary to determine whether dog fur can be safely incorporated into potting soil (particularly if used for the production of edible crops). Human hair waste can contain heavy metals and other toxic components (Alrobaian and Arida 2019; Galizia et al. 2024) that impact soil and plants (Fan et al. 2018) and possibly even humans who consume these plants (Li et al. 2012). However, even if dog fur is ultimately deemed unsafe for edible crops, it could replace some of the peat moss used extensively for the production of ornamental container plants. It seems highly unlikely that dog fur - collected from household pets that often live in close proximity to children - would present safety concerns in the context of ornamental plants. Finally, issues of scale will need to be investigated, including the amount of peat moss that could be reasonably displaced by the dog fur waste that is generated by commercial grooming salons.

Despite these uncertainties, our initial findings are extremely encouraging. Given that dog fur seems to increase both vegetative and reproductive structures, it may be beneficial in a wide range of applications spanning both edible and ornamental plant production. Overall, the evidence thus far suggests that dog fur has tremendous potential as an environmentally friendly potting soil component that can reduce the use of both peat moss and synthetic fertilizer.

## Methods

*Experimental setup*

The suitability of dog fur as a potting soil medium was evaluated with three crops commonly grown in containers: basil ( *Ocimum basilicum* ; standard unnamed variety), lettuce ( *Lactuca sativa* ; variety = Black-Seeded Simpson), and French marigold ( *Tagetes patula* ; variety = Sparky). Each crop species was subjected to three dog fur concentrations (by volume): 0%, 25%, and 50% dog fur. Dog fur, which was collected from the Petco grooming salon in Ashland, Virginia (United States), contained fur from various, unknown dog breeds, but the fur was mixed thoroughly prior to use to minimize the possibility of bias from certain fur types. In addition, to determine if surface contaminants (e.g. shampoo) influence plant growth results, half of the fur was triple-rinsed thoroughly with water (three sequential soakings) and left to dry before experimentation began. Thus, there were five treatment groups for each species: a) control (0% dog fur), b) 25% fur - unrinsed, c) 25% fur - rinsed, d) 50% fur - unrinsed, and e) 50% fur - rinsed.

Control plants were grown in Sungro “Professional Growing Mix” (MM830/3B RSi), and this same product was thoroughly mixed with dog fur for the 25% and 50% dog fur treatments (75% and 50% Sungro, respectively). The precise recipe for this standard potting mix is commercially protected, but the label indicates that it is composed of Canadian sphagnum peat moss (45-55%), composted bark, perlite, dolomite lime, a wetting agent, and an additive called “RESiLIENCE”, which contains silicon dioxide and is purported to increase root growth. Plant nutrient quantities are not provided.

All plants were grown in 4” plastic pots during the fall of 2024 in a controlled lab setting at Randolph-Macon College in Ashland, Virginia. Three seeds of the desired plant type were added to the center of each pot, which was then watered thoroughly and placed under a grow light on a 12-hour cycle. After germination, each pot was thinned to one plant to eliminate competition. Due to variable germination, final replicate numbers differed across species and treatments (Table 1). All pots received equal amounts of water every two days throughout, and the pots were frequently rearranged, randomly, to ensure that each experienced comparable conditions over time.

Table 1.&nbsp; Final sample sizes of each treatment level for each species.

**Table:** 

&nbsp;	0% fur	25% fur		50% fur	
&nbsp;	&nbsp;	unrinsed	rinsed	unrinsed	rinsed
Basil	5	5	5	5	5
Lettuce	10	10	9	7	7
Marigold	5	6	4	1	6

*Data collection*

Plants were allowed to grow until the largest individuals of each species began to outgrow their pots. Harvesting was done for basil on day 44, lettuce on day 45, and marigold on day 56. Two response variables (fresh weight and leaf color) were recorded for all three species, and some species-specific variables were also measured (see below). To guarantee reliable fresh weight values, all plants were thoroughly watered two hours prior to harvest, ensuring full hydration. Plants were cut at the soil line and the entire aboveground portion of each plant was weighed. Prior to harvest, a leaf color chart score (ranging from 1 to 6, with 1 representing a faded yellowish green and 6 representing a deep dark green) was assigned to each plant by selecting the single value that best matched the general, overall color of the plant (Fayaz et al. 2022). Additional species-specific variables, each collected immediately before harvest, consisted of plant height (basil and marigold), plant diameter (two perpendicular measurements; lettuce only), leaf count (>= 3 cm; lettuce and basil), and the number of open flowers and flower buds (marigold only).

*Data analysis*

To facilitate comparison across species, fresh weight values were standardized to the mean of the control group (0% fur) for each species. The resulting *fresh weight index* thus has a mean value of 1.0 for the control group of each species, and other values can be readily interpreted with respect to the control (for instance a value of 2.0 would indicate a fresh weight twice that of the control mean for that species). For lettuce, the two perpendicular diameter measurements per plant were averaged, and mean diameters were analyzed. For marigold, open flower and flower bud counts were summed, and this combined total was analyzed. All other response variables were subjected to analysis with the original raw values.

Due to uneven sample sizes (including a single replicate in one case), we did not analyze all five treatment groups simultaneously. Instead, we conducted two separate sets of analyses, the first using fur percent (0, 25, or 50% dog fur) as the explanatory variable (with unrinsed and rinsed groups pooled), and the second using rinse treatment (rinsed, unrinsed, or NA [no dog fur]) as the explanatory variable (with 25% and 50% dog fur groups pooled). We conducted separate analyses for each species, and - for fresh weight and leaf color (the two variables that were collected for all species) - we also performed analyses with all species combined. In all cases, we used Tukey HSD tests to determine which treatments were significantly different. All analyses were performed with the R Statistical Software (R Core Team 2024), and all figures were created with the *ggplot2* package (Wickham 2016).
